# Chestnut polysaccharides benefit spermatogenesis through improvement in the expression of important genes

**DOI:** 10.18632/aging.103205

**Published:** 2020-06-21

**Authors:** Shuai Yu, Yong Zhao, Fa-Li Zhang, Ya-Qi Li, Wei Shen, Zhong-Yi Sun

**Affiliations:** 1 Urology Department, Peking University Shenzhen Hospital, Shenzhen 518036, China; 2 Center for Reproductive Medicine, Peking University Shenzhen Hospital, Shenzhen 518036, China; 3 College of Life Sciences, Qingdao Agricultural University, Qingdao 266109, China; 4 Urology Department, Zaozhuang Hospital of Zaozhuang Mining Group, Zaozhuang 277100, China

**Keywords:** chestnut polysaccharides, spermatogenesis, gene expression, hormone

## Abstract

Recently there has been a continuing worldwide decrease in the quality of human spermatozoa, especially in spermatozoa motility and concentration. Many factors are involved in this decline, and great efforts have been made to rescue spermatogenesis; however, there has been little progress in the improvement of sperm quality. Chestnuts are used in traditional Chinese medicine; their major active components are chestnut polysaccharides (CPs). CPs have many biological activities but their effects on spermatogenesis are unknown. The current investigation was designed to explore the impact of CPs on spermatogenesis and the underlying mechanisms. We demonstrated that CPs significantly increased sperm motility and concentration (4-fold and 12-fold, respectively), and improved seminiferous tubule development by increasing the number of germ cells after busulfan treatment. CPs dramatically rescued the expression of important genes and proteins (STRA8, DAZL, SYCP1, SYCP3, TNP1 etc.) in spermatogenesis. Furthermore, CPs increased the levels of hormone synthesis proteins such as CYP17A1 and HSD17β1. All the data suggested that CPs improved the testicular microenvironment to rescue spermatogenesis. With CPs being natural products, they may be an attractive alternative for treating infertile patients in the future. At the same time, the deep underlying mechanisms of their action need to be explored.

## INTRODUCTION

During the past three decades, a growing number of people have been suffering from reproductive health issues leading to infertility. Many factors are involved in infertility, but in the male, a decrease in the quality of spermatozoa is a key factor. Many studies have revealed that environmental pollution and ionizing radiation played vital roles in the deterioration of spermatozoa quality (spermatozoa motility and concentration) [1–4]. Therefore, a feasible approach to protect fertilization capacity or to reverse any damage is necessary.

Recently, it has been found that polysaccharides have many biological functions. They pose anti-oxidative and anti-inflammatory functions through promoting the expression of antioxidant enzymes and decreasing the expression levels of inflammation factors [5–8], immunobiological activity [9, 10], and anti-cancer functions [11, 12]. Chestnuts are found throughout China and are used in traditional Chinese medicine [13]; they contain many nutrients that have several health benefits. Chestnut polysaccharides (CPs) include many monosaccharides such as glucose, rhamnose, arabinose, galactose, xylose, mannose, and fructose. CPs have been shown to have anticancer activity [14, 15], antifatigue effects [16], and antioxidative activity [17–21] via inhibiting tyrosinase and cancer cell proliferation; they also improve the ability of endurance and scavenge free radicals. Although CPs have many biological functions, their effects on the recovery of spermatogenesis are unknown.

Spermatogenesis takes place in the seminiferous tubules of the testis; it includes spermatocyte proliferation, spermatogonial differentiation into spermatocytes, spermatid production, and sperm maturation. Many factors are involved in regulating spermatogenesis such as genes, hormones, and epigenetic regulators [22–24]. The most important genes include *VASA*, *DAZL*, and *DMC1*. *VASA* is a germ cell marker important for germ cell proliferation and differentiation, and *VASA* mutation results in the cessation of germ cell differentiation [25]. *DAZL* serves as a gateway in oogenesis and spermatogenesis, and the abnormal expression of *DAZL* will affect the initiation of gametogenesis [26]. *DMC1* plays an important role in spermatogenesis, and its mutation leads to obstacles in male sterility [27].

Hormones such as testosterone and estrogen play essential roles in regulating spermatogenesis [28]. Many proteins such as cytochrome P450, cholesterol side-chain cleavage enzyme (CYP11A1), hydroxy-Δ-5-steroid dehydrogenase 3β-steroid Δ-isomerase 1 (HSD3β1), cytochrome P450 17α-hydroxylase/C17, and 20-lyase (CYP17A1) [29, 30] are involved in the synthesis of testosterone and estrogen.

Although CPs have been shown to be beneficial for human health, the effects on spermatogenesis and the underlying mechanisms are not understood. The aim of this study was to explore the means of CPs improve spermatogenesis and the underlying mechanisms.

## RESULTS

### CPs increased sperm motility and sperm concentration

**Figure 1:**
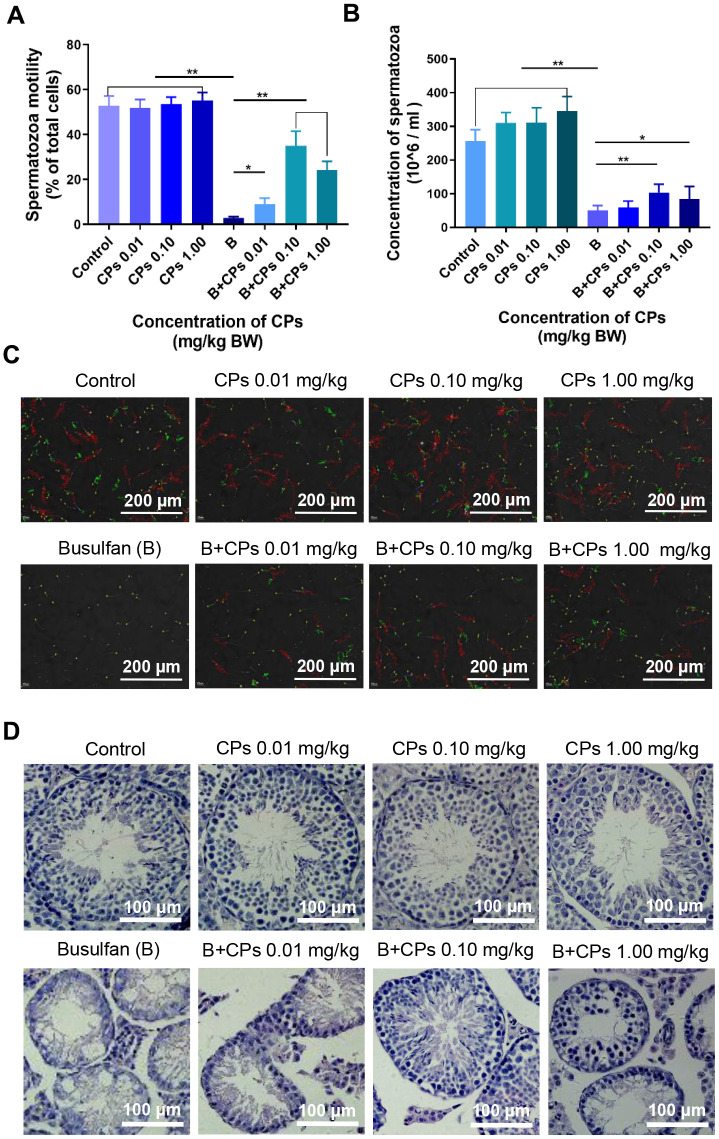
**Chestnut polysaccharides (CPs) improved sperm quality.** (**A**) Sperm motility (% of total cells; grade A + grade B). X-axis represents the treatment groups; Y-axis represents sperm motility. Data are represented as mean ± SEM, *P < 0.05, **P < 0.01. (**B**) Sperm concentration. X-axis represents the treatment groups; Y-axis represents sperm concentration (million/ml). Data are represented as mean ± SEM, *P < 0.05, **P < 0.01. (**C**) Photos of sperm quality. (**D**) Histopathology photos of mouse testes.

CPs alone did not change murine sperm motility (Figure 1A), however, sperm concentration was increased significantly (Figure 1B). Busulfan dramatically disrupted spermatogenesis by decreasing sperm motility and concentration almost to a level of infertility (Figure 1A–1C). However, busulfan plus CPs significantly increased sperm motility and concentration, especially in the B+CPs 0.10 mg/kg group (Figure 1A, 1B). Busulfan impaired spermatogenesis through decreasing the number of spermatogenetic cells and disrupting the structure of seminiferous tubules, as revealed by testicular histopathology (Figure 1D). CPs alone did not change the structure of the seminiferous tubules; however, busulfan plus CPs dramatically improved seminiferous tubules through an increase in the number of germ cells, especially in the B+CPs 0.10 mg/kg group (Figure 1D). Testicular histopathology confirmed the data for sperm motility and concentration. We then set out to explore how CPs improved spermatogenesis. The concentration of 0.10 mg/kg CPs produced a profound improvement, therefore this dose was used for further investigations. Body weights and organ indexes are shown in Table 1.

**Table 1:** Mouse body parameters.

	**Control**	**CP 0.01μg/kg**	**CP 0.10μg/kg**	**CP 1.00μg/kg**	**B**	**B+ CP 0.01μg/kg**	**B+ CP 0.10μg/kg**	**B+ CP 1.00μg/kg**
Body weight (g)	36.27±1.45	37.49±0.92	36.59±1.16	36.88±0.72	33.80±1.04	26.13±1.51^**^	30.72±1.03	31.54±1.00
Kidney index	1.65±0.052	1.67±0.04	1.63±0.04	1.68±0.03	1.83±0.06	1.50±0.05^*^	1.67±0.04	1.72±0.04
Spleen index	0.49±0.06	0.66±0.15^*^	0.39±0.03	0.44±0.05	0.36±0.02	0.61±0.08	0.39±0.02	0.38±0.01
Liver index	6.06±0.13	6.30±0.20	6.00±0.11	5.76±0.14	6.34±0.27	5.62±0.09	5.57±0.12^*^	5.73±0.13

Data is presented as mean ± SEM. * indicate a significant difference compared with B group (*P < 0.05*), ** indicate a significant difference compared with B group (*P < 0.01*). Index (organ weight/body weight).

Note: B=busulfan; CPs=Chestnut polysaccharides.

### CPs improved the expression of important genes involved in spermatogenesis in mouse testes

**Figure 2:**
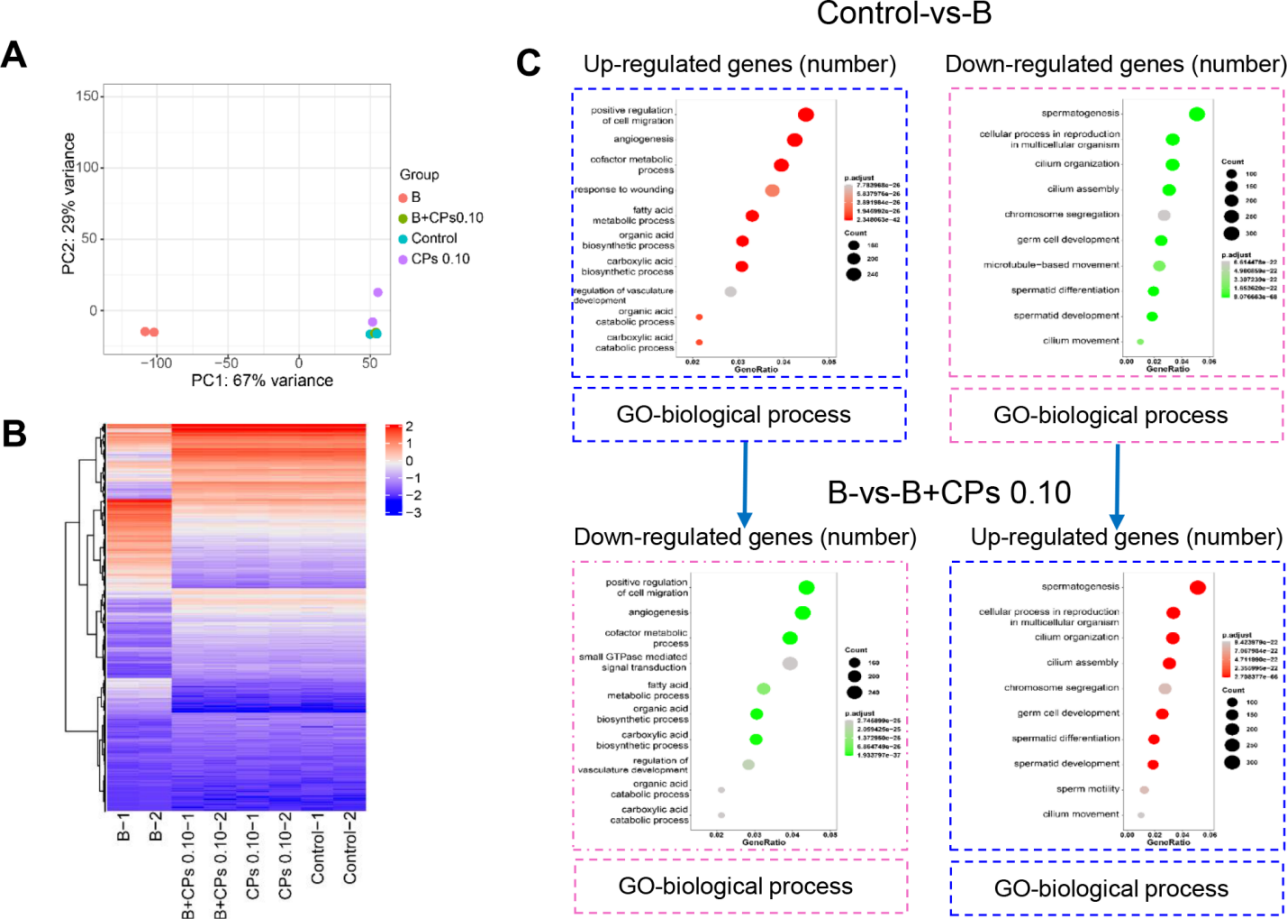
**Overview of the RNA-seq data for mouse testes.** (**A**) Principal components analysis (PCA). (**B**) The heatmap of differentially expressed genes (DEGs). (**C**) GO enrichment analysis of up-regulated and down-regulated genes in biological processes in the Control-vs-B and B-vs-B+CPs 0.10 groups, respectively.

First, testicular tissue transcriptomes were determined after busulfan and/or CPs treatments to search for gene expression patterns. Principal components analysis (PCA) showed that the busulfan and control groups were well separated, which suggested that the busulfan treatment produced profound effects on gene expression (Figure 2A). The B+CPs 0.10 mg/kg group overlapped with the control group, which suggested that the CP 0.10 mg/kg group recovered the gene expression that was changed by busulfan (Figure 2A). In total, 52 459 genes were found in the testicular tissues in the current investigation. A total of 15 738 genes were differentially expressed in the Control-vs-B group including 10 136 genes down-regulated and 5602 genes up-regulated. In addition, 13 796 genes were differentially expressed in the B-vs-B+CPs 0.10 mg/kg group including 4398 genes down-regulated and 9398 genes up-regulated (Figure 2B). The functions of these differentially expressed genes (DEGs) were displayed by GO analysis. In the comparison of the Control-vs-B group, the genes decreased by busulfan were enriched during spermatogenesis, germ cell development, and other sperm function related groups; the genes that were increased were in other functional groups (non-sperm related groups). But, in the comparison of the B-vs-B+CPs 0.10 groups, the genes increased by treatment B+CPs 0.10 were enriched during spermatogenesis, germ cell development, and other sperm function related groups, while the decreased genes were enriched in other functional groups (non-sperm related groups). The data suggested that treatment B+CPs 0.10 reversed the disruption of spermatogenesis caused by busulfan (Figure 2C).

**Figure 3:**
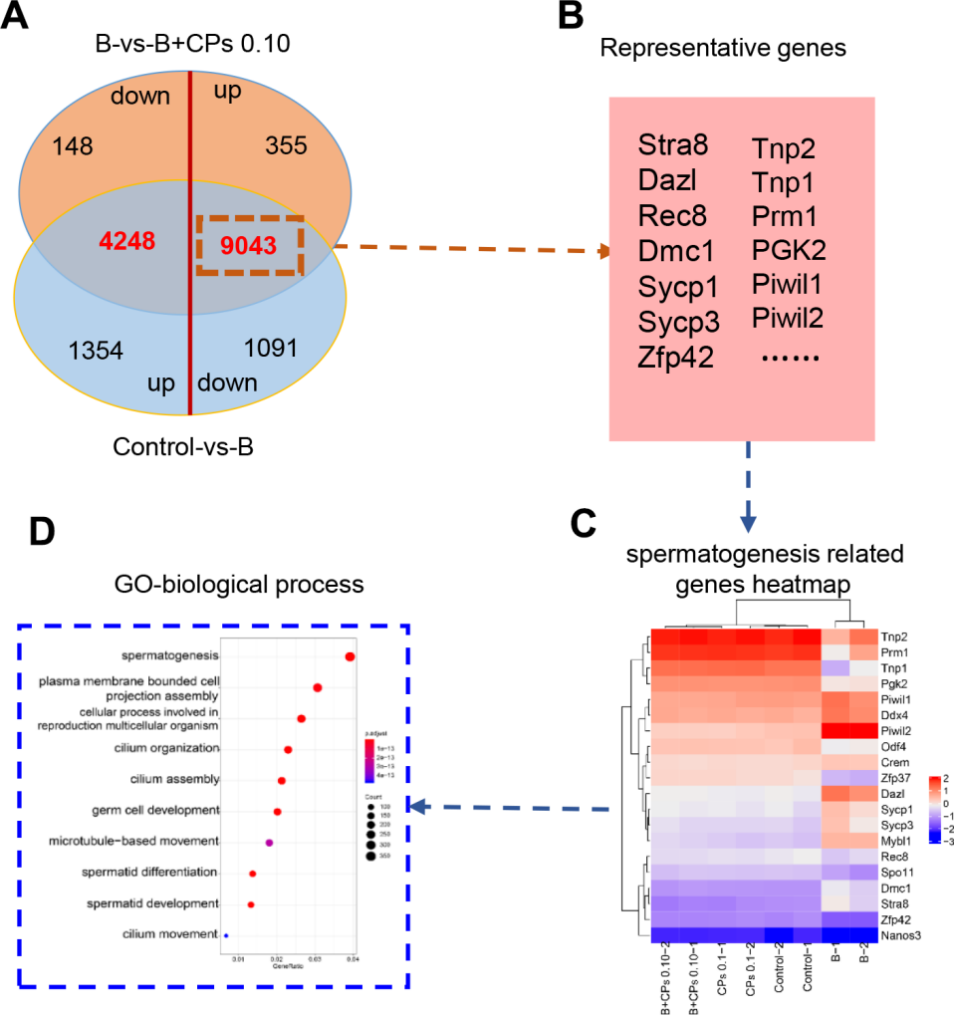
**Differentially expressed genes (DEGs) involved in spermatogenesis.** (**A**) The Venn diagram of up-regulated and down-regulated genes in the intersection of the Control-vs-B and B-vs-B+CPs 0.10 groups. (**B**) The representative genes in spermatogenesis. (**C**) The heatmap of 20 representative genes in spermatogenesis. (**D**) GO enrichment analysis of the DEGs in biological processes.

**Figure 4:**
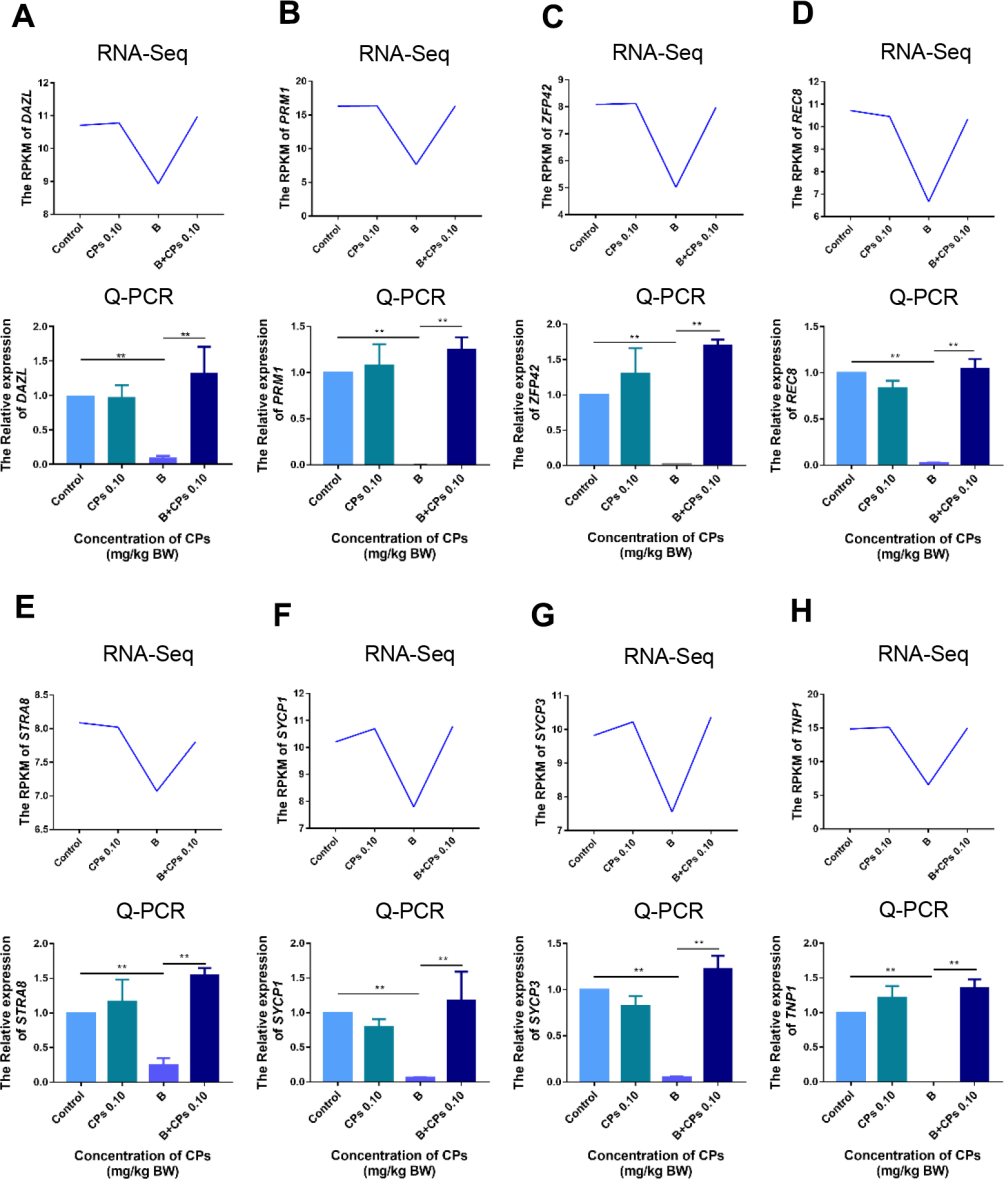
**The expression of important genes in spermatogenesis by RNA-seq, and confirmed by q-RT-PCR.** (**A**) *DAZL*; (**B**) *PRM1*; (**C**) *ZFP4*2; (**D**) *REC8*; (**E**) *STRA8*; (**F**) *SYCP1*; (**G**) *SYCP3*; and (**H**) *TNP1*. The results are presented as mean ± SEM, **P < 0.01.

Then we compared the DEGs in Control-vs-B and B-vs-B+CPs 0.10. It was interesting that most of the decreased DEGs in Control-vs-B were increased in B-vs-B+CPs 0.10; while most of the increased DEGs in Control-vs-B were decreased in B-vs-B+CPs 0.10 (Figure 3A). Spermatogenesis is a complex process involving many genes, such as *VASA*, *CREM*, *SYCP3*, *DAZL*, *REC8*, *PGK2*, *DMC1*, *SYCP1*, *STRA8*, and *ZFP42*. Most of the DEGs that were decreased in Control-vs-B while being increased in B-vs-B+CPs 0.10, were involved in spermatogenesis or reproduction, including *STRA8*, *DAZL*, *SYCP1*, *SYCP3*, *TNP1,* etc. (Figure 3B–3D and Supplementary Figure 1). Quantitative RT-PCR (qRT-PCR) was used to verify the RNA-seq data. The expression of eight DEGs was confirmed and the results revealed a similar trend for RNA-seq and qRT-PCR (Figure 4).

**Figure 5:**
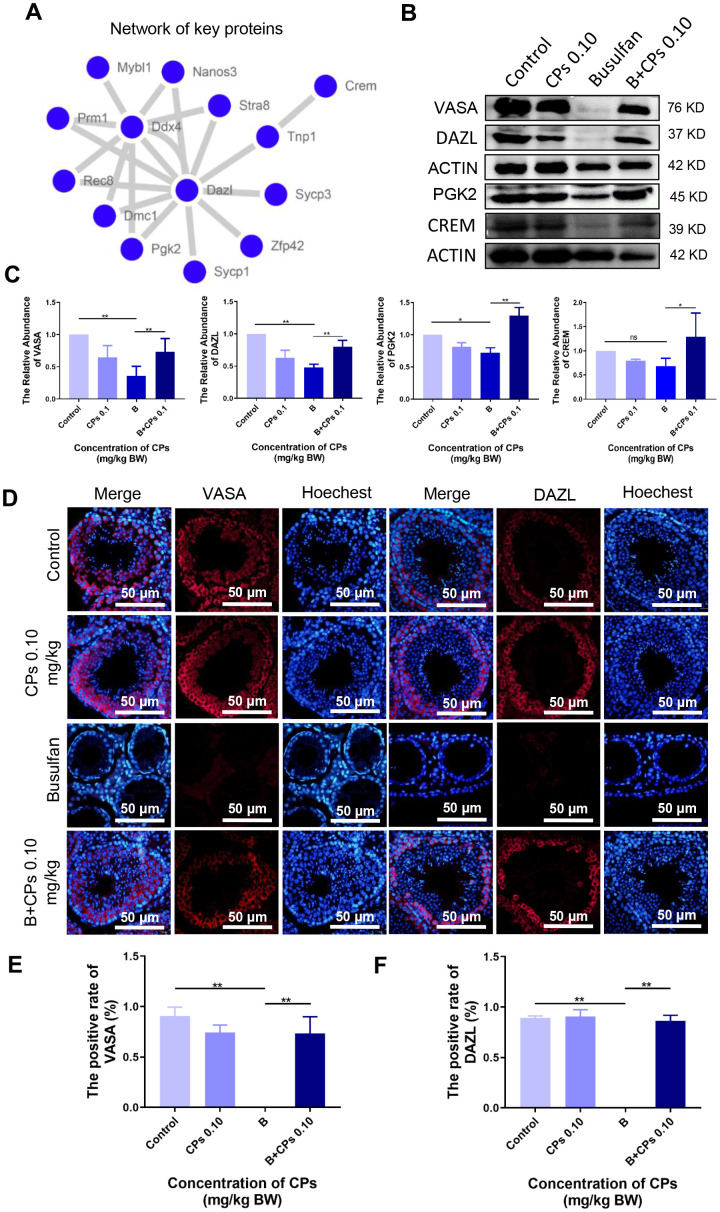
**The expression of important proteins in spermatogenesis.** (**A**) The network of important genes/proteins in spermatogenesis. (**B**) The protein levels of VASA, DAZL, PGK2, and CREM using Western blot analysis. (**C**) The relative abundance of VASA, DAZL, PGK2, and CREM in different groups, ns is stand for no difference, *P<0.05, **P < 0.01. (**D**) The immunofluorecence staining images of VASA and DAZL. (**E**) The quantitative data for VASA in immunofluorecence staining. The results are presented as mean ± SEM, **P < 0.01. (**F**) The quantitative data for DAZL in immunofluorecence staining. The results are presented as mean ± SEM, **P < 0.01.

Furthermore, the protein levels of important genes involved in spermatogenesis were determined in the current investigation. Many of these proteins formed a network to regulate spermatogenesis (Figure 5A). Busulfan alone decreased the protein levels of DAZL, PGK2, CREM, and VASA, while B+CPs 0.10 increased these protein levels as revealed by Western blot analysis (Figure 5B, 5C). Immunohistochemistry (IHC) confirmed the protein changes during spermatogenesis. The number of positive cells staining for VASA and DAZL were decreased by busulfan while they were increased by B+CPs 0.10 as seen by IHC (Figure 5D–5F) which confirmed the Western blot analysis data. The data in this section suggested that CPs rescued mouse spermatogenesis through an improvement in the levels of relevant genes and proteins.

### CPs increased the level of proteins involved in hormone production

**Figure 6:**
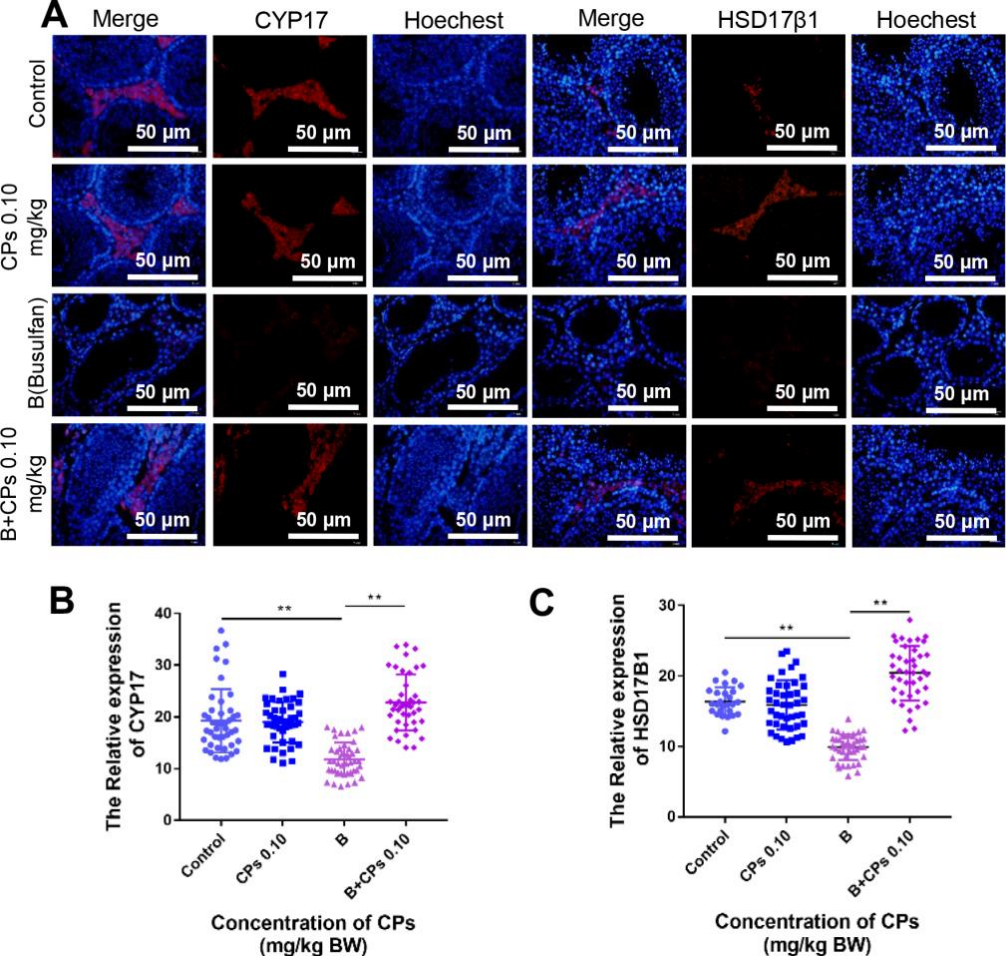
**Effects of CPs on hormone synthesis exposed to busulfan.** (**A**) The representative images of HSD17β1 and CYP17 in the Control, CPs 0.10, B, and B+CPs 0.10 groups. (**B**) The fluorescence intensity analysis of CYP17 in different groups (Control: 19.28 ± 0.9276, CPs 0.10: 18.97 ± 0.5916, B: 11.8 ± 0.4849, B+CPs 0.10: 22.8 ± 0.8389) (**C**) The fluorescence intensity analysis of HSD17β1 in different groups (Control: 16.38 ± 0.3876, CPs 0.10: 15.88 ± 0.537, B: 9.892 ± 0.2834, B+CPs 0.10: 20.42 ± 0.6048). The results are presented as mean ± SEM. **P < 0.01.

Testosterone and estrogen play vital roles in spermatogenesis and many proteins (or enzymes) are involved in the production of these hormones. We determined these proteins by IHC and found that the levels of CYP17A1, and HSD17β1 were decreased by busulfan while elevated by B+CPs 0.10 (Figure 6A–6C). The data here suggested that CPs 0.10 may improve the local hormones to enhance spermatogenesis.

## DISCUSSION

Chestnuts have been used as a traditional food and medicine for millennia in China due to their health advantages [31]. CPs, key substances in chestnut, have been investigated extensively; they are known to inhibit cancer cell proliferation, enhance endurance performance, and to reduce ROS levels [14–21]. However, the effects of CPs on the recovery of spermatogenesis are not understood. Therefore, in the present study we aimed to investigate the rescuing effects of CPs on spermatogenesis after busulfan treatment. We found that busulfan disrupted spermatogenesis by decreasing the motility and concentration of mouse sperm, through reducing the expression of genes and proteins involved in spermatogenesis, which was similar to the findings of previous studies [32–35]. On the other hand, CPs increased the motility and concentration of mouse sperm, especially at a treatment level of 0.10 mg/kg.

Subsequently, we explored the underlying mechanisms of CPs in rescuing mouse spermatogenesis. Gene expression within testicular samples was determined by RNA-seq analysis. It was interesting to notice that 9043 DEGs were decreased by busulfan while increased by CPs, and 4248 DEGs were increased by busulfan while decreased by CPs. GO enrichment analysis revealed that these 9043 DEGs, that were decreased by busulfan while being increased by CPs, were enriched in spermatogenesis related functional pathways. But the 4248 DEGs, those were increased by busulfan while decreased by CPs, were enriched in other functional pathways (non-sperm related groups). The data indicated that CPs may regulate gene expression machinery to improve spermatogenesis.

Moreover, we explored the effect of CPs on hormone production pathways in mouse testes, because of the critical role that hormones play in spermatogenesis [28, 36, 37]. CPs increased the protein levels of CYP17A1 and HSD17β1 which were decreased by busulfan. CYP17A1 and HSD17β1 are important for the synthesis of hormones such as testosterone and estrogen, which are essential for spermatogenesis. The data suggested that CPs benefit the testicular microenvironment to improve spermatogenesis.

In conclusion, the results of this study suggested that CPs improved spermatogenesis by increasing sperm motility and concentration. The underlying mechanisms involved CPs regulation of gene expression machinery to increase the expression levels of genes and proteins involved in spermatogenesis and to improve the testicular microenvironment to rescue spermatogenesis. A decrease in the quality of spermatozoa is a key factor that caused infertility. Therefore, looking for a feasible approach to protect fertilization capacity or to reverse any damage in testes is necessary. CPs being as natural products, they may be an attractive alternative method for treating infertile patients in the future.

## MATERIALS AND METHODS

### Mouse experimental environment

Male ICR mice were purchased from the Beijing Vital River Laboratory Animal Technology Co., Ltd (Beijing, China). The mice were fed in constant temperature (22-23°C) rooms with a 12 h light: 12 h dark photoperiod and had free access to food (chow diet) and water throughout the period of study. All procedures used in this study were in accordance with the guidance of the Ethics Committee of Qingdao Agricultural University.

### Mouse treatments and sample collection

Busulfan was used as a model compound to create an infertile animal model as it has been frequently used in recent published articles [38, 39]. CPs were brought from Wo Te Lai Si biotechnology co., LTD (Lan Zhou, China). We set out to examine the remedial efficacy of CPs on busulfan-exposed murine spermatogenesis and to examine the underlying mechanisms. Male mice under treatment were exposed to busulfan at a concentration of 40 mg/kg and CPs treatment via oral gavage. The concentrations of CPs used in this investigation were 0.01-1.00 mg/kg. The different concentrations of CPs solutions were freshly prepared every day in ultrapure water. There were 8 treatments (10 mice/treatment): (1) vehicle control (ultrapure water); (2) CPs 0.01 mg/kg (CPs 0.01); (3) CPs 0.10 mg/kg (CPs 0.10); (4) CPs 1.00 mg/kg (CPs 1.00); (5) busulfan (B); (6) busulfan + CPs 0.01 mg/kg (B+CPs 0.01); (7) busulfan + CPs 0.10 mg/kg (B+CPs 0.10); (8) busulfan + CPs 1.00 mg/kg (B+CPs 1.00). The volume of fluid used for the oral gavage was 0.10 ml/mouse/day. In this study we chose 3-week-old mice, the gavage took place every morning and lasted for 5 weeks. After 5 weeks, the mice were humanely slaughtered and we collected tissue samples for analysis such as testis, blood, and sperm.

### Evaluation of sperm motility and concentration

Sperm motility was analyzed during sample collection. Sperm were collected from the epididymis then cultured in DMEM/F12 (Gibco, 8119172, U.S.A.) with 10% FBS (Gibco, 10099-141, USA) medium and incubated at 37.5°C on a heated stage for 5 min, counting boards were prepared in advanced. Then samples were placed in a counting chamber [35] and sperm motility and concentration were analyzed using an SCA sperm class analyzer. We used WHO standards for the analysis of sperm motility: sperm progressing at above 22 μm/s were defined as grade A; the curvilinear velocity of sperm under 22 μm/s and greater than 5 μm/s were defined as grade B; the curvilinear velocity of sperm under 5 μm/s were defined as grade C and immotile sperm were defined as grade D [40–42]. Concentration was evaluated during motility and sperm were diluted with medium during the process of evaluation [40, 41].

### RNA extraction and RNA-seq and bioinformatics analysis

Total RNA was extracted from collected testis samples using a kit (Thermo Fisher 12183018A, USA) and according to the manual instructions [43]. Sequencing was conducted using an Illumina HiSeq2000 system (Novogene Co. Ltd., Beijing, China). FastQC (v0.11.8) and Fastp (v0.19.5) were adopted to assess the quality of the raw data and to remove low quality data, adapter, and poly-N sequences, respectively. Clean data were used in subsequent analyses [44]. DEGs were analyzed using the DESeq2 package. R Bioconductor/cluster Profiler package and Gene Ontology (GO) were used to analyze DEG functional profiles [45]. The Search Tool for the Retrieval of Interacting Genes/Proteins (STRING) was used to predict protein interaction network [46].

### Quantitative real-time PCR

Total RNA was reverse transcribed using the TransScript One-Step gDNA Removal Kit and cDNA Synthesis Kit (TransGen, AT311-03, Beijing, China). To evaluate the changes in mRNA level, we conducted quantitative real-time PCR (qRT-PCR) using transcribed cDNA. qRT-PCR was conducted using a Light Cycler real-time PCR instrument (LC480, Roche, Basel, Switzerland) using Light Cycler SYBR Green I Master Mix according to the manufacturer’s instructions. A reaction system volume of 24 μl was used in this study containing 2 μl cDNA, 12 μl of SYBR green master mix, 1.2 μl of primer mix including forward and reverse primer genes, and 8.8 μl of RNAase-free water. The PCR conditions of a previous study were followed [47]. We quantified actin as an internal reference when analyzing gene expression. Relative expression abundance was calculated using the method given in a previous study [48]. Gene primer sequences are listed in Supplementary Table 1.

### H&E and immunofluorescence

Collected testes were fixed in 4% paraformaldehyde and kept in a refrigerator at 4°C overnight, then subsequently stored in different concentrations of dehydrating solutions. The dehydrated testicular samples were then embedded in paraffin and the resulting paraffin blocks were sectioned at 5 μm thickness following standard histological procedures. Sections were stained with H&E, following the procedure of a previous study [49, 50]. Testicular sections were also used for IHC; the sections were prepared and subjected to xylene and ethanol solution followed by antigen retrieval. Sections were then blocked with blocking buffer [3% bovine serum albumin (BSA, Solarbio, A8020, Beijing, China), 10% normal goat serum in TBS buffer] at room temperature for 30 min. Each section was incubated with primary antibodies (Supplementary Table 2) and secondary antibodies (Beyotime, A0516, Nantong, China) then sections were imaged under an Olympus fluorescence microscope (Olympus, BX51, Tokyo, Japan) [51].

### Western blot analysis

Testicular proteins were extracted using the process described below. Samples were submerged in cold RIPA buffer and a crusher was used to treat the samples twice at a cold temperature. The protein lysates were centrifuged at 10000 rcf/min for 10 min then the supernatant was collected and added to SDS-loading buffer, and boiled for 5 min in water till the protein was completely denatured. Protein samples were mixed evenly before loading onto 10% SDS polyacrylamide electrophoresis gels and processed at 100V for 1.5 h; the specific time depended on the electrophoresis situation. The proteins on the gels were transferred to a polyvinylidene fluoride membrane. Subsequently, membranes were blocked with skimmed milk or 5% bovine serum albumin at a cold temperature for at least an hour, followed by 3 washes with Tris-buffered saline, 0.1% Tween 20 (TBST). We then used primary antibodies to incubate membranes in the refrigerator overnight at 4°C. On the second day, membranes were washed 3 times with TBST and incubated with the HRP-labeled secondary antibody (Beyotime, A0208, Nan tong, China) for 1 h at room temperature. After 3 washes with TBST, the membranes were imaged [51]. The images were analysed using the AIC. Alpha View software.

### Statistical analysis

All experiments were repeated at least 3 times and results were expressed as the mean ± SEM. SPSS software one-way analysis of variance (ANOVA) following by LSD multiple comparison test was used for data analysis and we defined *P < 0.05* as a significant difference, while *P < 0.01* was a highly significant difference.

## Supplementary Material

Supplementary Figure 1

Supplementary Tables
