# Supplementary material for: Chestnut polysaccharides benefit spermatogenesis through improvement in the expression of important genes
Source: Aging (Albany NY). 2020 Jun 21;12(12):11431–45. doi: 10.18632/aging.103205 (PMC7343452; doi:10.18632/aging.103205)
Supplement: Supplementary Figure 1 [file aging-12-103205-s002..pdf]

## SUPPLEMENTARY FIGURE

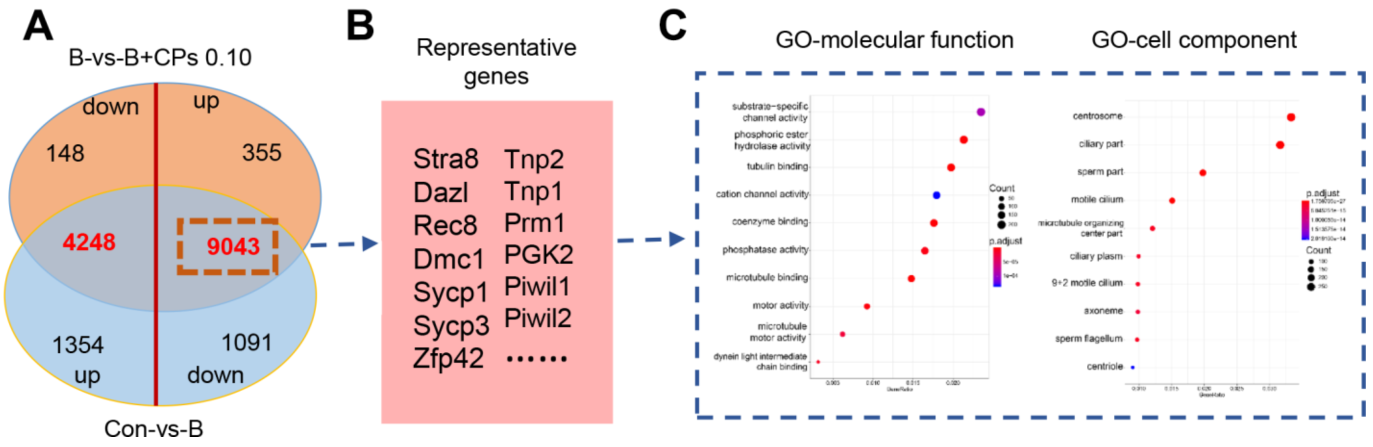


**Supplementary Figure 1. DEGs involved in spermatogenesis.** (**A**) The Venn diagram of up-regulated and down-regulated genes in the intersection of the Control-vs-B and B-vs-B+CPs 0.10 groups. (**B**) The representative genes in spermatogenesis. (**C**) GO enrichment analysis of the DEGs in molecular function and cellular components in the intersection of the Control-vs-B and B-vs-B+CPs 0.10 groups.
