# Supplementary material for: Chestnut polysaccharides benefit spermatogenesis through improvement in the expression of important genes
Source: Aging (Albany NY). 2020 Jun 21;12(12):11431–45. doi: 10.18632/aging.103205 (PMC7343452; doi:10.18632/aging.103205)
Supplement: Supplementary Tables [file aging-12-103205-s001..pdf]

SUPPLEMENTARY TABLES

Supplementary Table 1. Primers used for quantitative RT-PCR.

Genes	GenBank	Forward primer sequences	Reverse primer sequences
β-actin	NM_007393.5	ACCTCACTGACTACCTGATGA	CCACATAGCACAGCTTCTCTT
DAZL	NC_000003.12	TCCTTGACTTGTGGTTGCTG	CCACCTTCGAGGTTTTACCA
PRM1	NC_000082.6	AGCAAAAGCAGGAGCAGATG	CTTGCTATTCTGTGCATCTAG
ZFP42	NC_000074.6	TCCATGGCATAGTTCCAACAG	TAACTGATTTTCTGCCGTATGC
REC8	NC_000080.6	GGTAAAGACCTGCGAGGAAA	GCGGAGAGATAGAGGGAGAA
STRA8	NC_000072.6	GTTTCCTGCGTGTTCCACAAG	CACCCGAGGCTCAAGCTTC
SYCP1	NC_000069.6	CTCAAGGAAACCTGTGCTAGAT	TGCACACGAAGTTCCTCAA
SYCP3	NC_000076.6	AGCAGAGAGCTTGGTCGGG	TCCGGTGAGCTGTCGCTGTC
TNP1	NC_000067.6	AGCCGCAAGCTAAAGACTCA	CTCTCTTGACGCCCTTGTGA

Supplementary Table 2. Information on the antibodies used in this paper.

Antibodies	Vendor	Dilution
HSD17β1 (IF)	Bioss (bs-6603R)	1:150
CYP17A1 (IF)	Bioss (bs-6695R)	1:150
PGK2 (WB)	Sangon (D221803)	1:500
CREM (WB)	Sangon (D152356)	1:500
VASA (IF/WB)	Abcam(ab13840)	1:150/1:1000
DAZL (IF/WB)	Abcam (ab2920)	1:150/1:1000
β-ACTIN (WB)	Abcam (ab3280)	1:1000
